# Active walking in broiler chickens: a flagship for good welfare, a goal for smart farming and a practical starting point for automated welfare recognition

**DOI:** 10.3389/fvets.2023.1345216

**Published:** 2024-01-08

**Authors:** Marian Stamp Dawkins

**Affiliations:** Department of Biology, University of Oxford, Oxford, United Kingdom

**Keywords:** smart farming, broiler chickens, welfare assessment, image processing, optical flow

## Abstract

Automated assessment of broiler chicken welfare poses particular problems due to the large numbers of birds involved and the variety of different welfare measures that have been proposed. Active (sustained, defect-free) walking is both a universally agreed measure of bird health and a behavior that can be recognized by existing technology. This makes active walking an ideal starting point for automated assessment of chicken welfare at both individual and flock level.

## Introduction

Smart technology is increasingly being used to monitor and manage the keeping of farm animals and has the potential to improve both efficiency and animal welfare (1–8). Use of smart technology is particularly advanced in the dairy sector, where automated monitoring has contributed to animal welfare by enabling each animal to have its own individualized diet and medical treatment (9).The practical application of smart technology to commercial poultry farming, however, raises somewhat different problems from those involving large animals. Firstly, there is the problem of the very large numbers of animals involved. In dairy farming, there are relatively few animals on each farm and each one contributes a significant proportion of the economic output of the whole herd, making it financially worthwhile for the health of each individual cow to be monitored and adjustments made to suit her own individual needs and welfare (9, 10). In commercial poultry farming, by contrast, the economic unit consists of thousands or even millions of chickens. 20,000–50,000 in one house is common for broiler (meat) birds and there are usually several houses on one farm and up to 7 flock cycles each year. The consequence of this is that the economic value of each individual chicken is small compared to that of the enterprise as a whole. As a result, management decisions such as when to apply medication, change the light regime, diet or drinker height are taken not for the benefit of the individual bird but for the average of the whole flock. Furthermore, poultry producers specify welfare outcomes such as mortality or hock burn not bird by bird but as percentage outcomes of a whole flock. In other words, current poultry production operates at flock level and would seem to need its own flock level technology.

This raises a second problem which is that welfare is an individual matter, not a property of a flock or herd. It is individual animals that suffer and feel pleasure or pain (11), so that flock level measures alone are not enough to guarantee good poultry welfare. Here smart technology has the potential to make a revolutionary contribution to improving broiler chicken welfare. Precision crop agriculture has already shown the advantages of applying treatments such as fertilizer or irrigation to specific parts of a field or even individual plants rather than treating the whole field as a single entity (12). Precision poultry farming similarly has the potential to give greater emphasis to the welfare of the individual bird than has so far been possible, for example, by alerting the farmer to the location of an injured or lame bird or to an area of a house where there is a potential problem such as smothering or over-crowding. Houses containing many thousands of birds would no longer have to be treated as a single unit but as flocks of many individuals, experiencing different conditions and having different welfare outcomes. This would enable greater focus on the welfare of individual animals than either farmers or machines are currently able to do. The technical problems of achieving this in practice, however, are considerable. Separating one bird out of a flock of thousands of identical white birds that, unlike plants, are constantly moving, merging and dispersing, in addition to having to operate in difficult conditions such as low light levels, pose major image processing problems that have yet to be fully overcome. Sound and thermal imaging pose similar problems of distinguishing birds from background and from each other.

The third problem is which welfare measure or measures to use. Attempts to capture what is meant by good welfare such as the Five Freedoms (13) and the Five Domains (14) have played an essential role in setting welfare goals that can be agreed by producers, scientists and the general public but they are over-arching aspirations, not detailed instructions on how to measure welfare in practice (15). For use on real farms, they need to be translated into actual measurements that can be used by human auditors or turned into algorithms for implementation by machine. This is not easy. The Welfare Quality® protocol for broiler chickens (16), for example, divides welfare into good feeding, good housing, good health and “appropriate behavior” and then has a list of different measurements that need to be made for each category, such as plumage cleanliness and litter quality for good housing and absence of hock burn and breast blisters for good health. Filling such a checklist takes considerable time, often involves subjective judgements and may alter the birds’ behavior by the presence of an observer (17) so that automating the process of welfare assessment would be a major advantage.

Although considerable progress has been made in automating welfare assessment of broiler chickens, it is still not in widespread commercial use. I here argue that this is because the problems outlined above still pose considerable barriers to implementation and that these can best be overcome by focusing (at least initially) on a key measure of welfare that has two properties: (i) it is universally agreed to be a major and necessary component of chicken welfare and (ii) it is distinctive enough to pose minimal technological problems for recognition in large flocks of chickens on commercial farms.

The behavior that best fits both these requirements is “active walking”. Active or sustained walking (where a bird walks continuously and with regular strides for a specified time) is not a complete measure of everything that everyone might want to include in the definition of good welfare but it is a sign of a healthy bird and is linked to many other components of good welfare. It is also distinctive and relatively easy for a machine to recognize. It is therefore ideally suited as a starting point for automated welfare recognition, a foundation to which more welfare measures can later be added as our future knowledge base grows and more sophisticated analytic techniques become widely adopted (18, 19).

## Active walking as a flagship welfare indicator

Although “welfare” has many components and means different things to different people (20), lameness or difficulty in walking is widely acknowledged to be one of the major welfare issues for broiler chickens (21, 22). Lameness limits the range of behavior animals can perform, including gaining access to food and water (23, 24) and lame birds show evidence of being in pain when they try to walk (25–27). Difficulty in walking is also often associated with a variety of other adverse welfare indicators such as hock burn, footpad dermatitis and dirty feathers (17, 28, 29) [but see (30)] as well as increased likelihood that a bird will be dead on arrival at the abattoir or be rejected as unfit for human consumption (30, 31). In addition to being a welfare problem on its own, lameness thus serves as a flagship indicator for a whole range of other welfare and production issues.

Conversely, the ability to move freely and without limping is a hallmark of health – the net output of a healthy, well-functioning body. Since difficulty in walking can have many different causes (foot ulcers, strained muscles, broken bones, infected joints etc.) (32, 33), healthy walking suggests health in all the component parts, including the overall mood or health of the whole individual. An animal that walks actively and freely has thus passed a kind of generalized health test that has included a test of many different body components and its activity may itself help to reduce lameness (34). An animal that chooses to walk actively of its own accord has also shown that it is motivated to move, suggesting that active walking is additionally a measure of the positive affective aspects of welfare that go beyond physical health (29).

Walking ability in broiler chickens is frequently assessed using either a 6-point (35) or a 3-point (36) gait scoring system. A group of birds is separated from the main flock and then released either singly or in small groups into an area where their walking is observed either for a set time, such as 15 s, or over a set distance. Some birds have to be encouraged to walk for this long as the aim of gait scoring is to establish a bird’s ability to walk when it has to, not its motivation to walk. Each bird is then assigned a gait score between 0 (normal walking) and 5 (or 3) for a bird that is unable to walk. With trained observers, gait scores have been shown to correlate well with more objective tests such as how quickly a bird can walk a given distance with or without obstacles (37, 38) and how long it remains standing when placed in shallow water – the “latency to lie” test (37, 39, 40). Gait scoring by human observers has the disadvantages that it is subjective, very time consuming (scoring at least 100 individual birds per flock is recommended (35, 41)), is intrusive in that it involves catching or penning birds and even then gives only a snapshot of the walking ability of a small proportion of the flock at one particular time. Automating the assessment process to give an objective, continuous measure of walking ability in large numbers of birds and showing how it changes throughout life would therefore enable information on walking ability to be collected on a much wider scale and so have the potential to improve the welfare of the billions of broiler chickens currently raised for human food across the world.

The process of automating the measurement of gaits can be achieved in different ways, most commonly by analysis of visual images from video or cctv where lame birds are detected by abnormalities of their body posture and movement (42–48). Other methods that have been used to analyze movement and can potentially measure gait include ultra-wide-band (UWB) tags (49–51) and accelerometers attached to individual birds (52, 53).

Most studies involving visual images have been carried out on small groups in pens rather than on commercial flocks and often involve assessing birds one at a time in specially built apparatus (48, 54). They thus overcome one of the problems with gait scoring – its subjectivity – but do not address the more serious issues of having to catch the birds, being very labor-intensive, and giving only a brief snapshot view of welfare on one occasion. This limits their usefulness to commercial broiler production. Busy farmers do not have time to apply tests that involve separating birds from the rest of the flock or putting them into special apparatus to measure their walking ability. There needs to be a simple, inexpensive way of gathering information from large numbers of birds on commercial farms as they go about their daily lives.

## Automated recognition of active walking at flock level

Flock behavior can be measured in various ways including sound (55, 56), thermal imaging (57) and visual images from cctv or video cameras (43, 58–61). Analyses of visual images starts by detecting movement, most commonly done measuring optical flow which involves comparing the patterns of light and dark in a series of images that have small time steps between them such as a sequence of video frames. Each frame is divided into a set of hundreds of tiny points or pixels which can be any shade of grey between black and white. By comparing the greyness or brightness of the pixels in one frame with the brightness of those same pixels in successive frames, any differences will reveal what has changed between the frames and therefore what has moved, how much it has moved and in what direction.

Using this approach, the overall level of activity shown by commercial chicken flocks has been shown to be correlated with walking ability as measured by human observers recording gait scores from a sample of individual birds. Flocks with higher overall mean levels of movement return lower (ie. better) gait scores than flocks with lower overall mean levels of movement and higher (i.e., worse) gait scores (43, 58–63). Furthermore, there is also a correlation between the mean level of optical flow in a flock and performance of individual birds in a latency-to-lie test: birds able to remain standing for the longest times come from flocks with the highest mean level of movement (64). However, the mean level of movement shown by a flock is only one possible measure of activity and does not distinguish between movement due to active walking and that produced by other kinds of behavior, nor does it provide any information about the numbers of birds that are walking with different degrees of proficiency. By using additional statistical descriptors of basic optical flow data, a more detailed picture of broiler chicken behavior can be obtained. Specifically, by using the skew and kurtosis of the movement distribution as well as the mean level of activity much more information about active walking can be obtained and a link can be made between flock and individual behavior (58, 59, 64).

As an illustration of what this means in practical terms, the descriptive statistics of the movement distribution for 18 commercial flocks in Switzerland for 1 day (day 28 of life) are shown in Table 1 (65). The distribution has a highly positive (left-hand mode) skew, coupled with a highly positive (right-hand tail) kurtosis, showing that most of the flock’s movement is clustered towards the low end of the movement distribution with a few outlier time intervals showing much more movement than average. This corresponds well to actual observations made inside broiler houses that broiler chickens spend up to 90% of their time sitting and only about 10% actively moving about (24, 66, 67).

**Table 1:** Descriptive statistics of optical flow from 18 broiler flocks collected from commercial farms in Switzerland on day 28 (65).

Mean	Variance	Skew	Kurtosis
0.24 (±0.24)	0.22 (±0.21)	4.76 (±4.77)	30.11 (±3.25)

A direct link between these flock level statistics and active walking by individual birds was shown by comparing optical flow output with detailed frame-by-frame observations of behavior from the same video recordings (68). With a definition of an actively walking bird as one that walked continuously for at least 10 s, there was a significant correlation between the number of birds walking actively in a given 15 min period and the mean, skew and kurtosis of optical flow during that same period of time. The more actively walking birds in a sequence, the higher the mean optical flow and the lower the skew and kurtosis. It appeared that it was specifically active walking that was responsible for these results, since there were no correlations between skew and kurtosis and either sitting or walking when these were measured by instantaneous scan sampling. Broiler chickens frequently shuffle, stand up or even walk a few steps before sitting down again but it was the prolonged active walking – a much higher level of movement – that the optical flow algorithm was picking up. The skew and the kurtosis were thus acting as “active walker detectors”, showing whether actively walking birds were unusual in a flock (high skew and kurtosis) or more common (lower skew and kurtosis).

This shows the importance of using a combination of optical flow variables, rather than single variables. A flock with no active walkers and one where all the birds are actively walking would both have a low skew and a low kurtosis, but would be distinguishable by a major difference in the overall mean level of movement, which therefore provides one measure of flock health. The skew and kurtosis show whether active walking is rare or common within that flock and thus provide another measure of flock health. Healthy flocks have many active walkers while in less healthy flocks, active walking is a rarity. The statistics of welfare therefore indicate that high welfare, healthy flocks have a both high mean optical flow (due to high levels of activity in a high percentage of the flock) and a low skew and kurtosis (due to high levels of activity being the norm for that flock).

Further direct evidence for the role of skew and kurtosis of optical flow in detecting active walking is that individual birds from home flocks with lower skew and kurtosis of optical flow perform better (faster) in runway tests with or without obstacles than birds from flocks with higher skew and kurtosis (64).

## Discussion

The potential of smart farming technology to improve the welfare of broiler chickens has not yet been fully realized, both because of the lack of agreement on how to measure their welfare and also because of the technical difficulties of working with such large numbers of near-identical animals. This paper shows that one interim solution to both these problems is the same: start simple. As far as defining welfare is concerned, an output that is universally agreed to be important is a good starting point, even if it does not include everything that everyone might mean by “good welfare”. As far as the technical issues are concerned, an output that is easy for a machine to recognize without leading to too many false positives is also a rational starting point.

Active walking meets both these criteria. If a broiler chicken walks actively for a set period of time, it has effectively passed a generalized health test and so provided positive evidence for an important component of good welfare. Active walking can also be measured with current technology because, being such a conspicuous behavior, it is less subject to the image processing errors that still beset the recognition of other less distinctive behaviors and it has the further advantage of being measurable at both flock and individual level. However, a serious limitation is that there is more to good welfare than just physical health and more to good health than just ability to walk. It would therefore be a mistake to see active walking as the only welfare measure we need to make, but it is a realistic first goal, one that could be implemented in the near future with great benefit to both birds and producers. Once this first goal has been achieved and been successfully applied to commercial farms, it will be important to move on to more ambitious goals by developing technology that focusses even more on individual birds and that incorporates a much wider range of welfare measures.

## Data availability statement

The original contributions presented in the study are included in the article/supplementary material, further inquiries can be directed to the corresponding author.

## Author contributions

MD: Conceptualization, Funding acquisition, Project administration, Supervision, Writing – original draft, Writing – review & editing.
